# A revision of Japanese species of the genus *Psammoecus* Latreille (Coleoptera, Silvanidae)

**DOI:** 10.3897/zookeys.403.7145

**Published:** 2014-04-17

**Authors:** Takahiro Yoshida, Toshiya Hirowatari

**Affiliations:** 1 Entomological Laboratory, Graduate School of Bioresource and Bioenvironmental Sciences, Kyushu University, Fukuoka, 812–8581 Japan; 2 Entomological Laboratory, Faculty of Agriculture, Kyushu University, Fukuoka, 812–8581 Japan

**Keywords:** Taxonomy, Cucujoidea, Japan, four new species, misidentification, redescription, taxonomic key

## Abstract

Japanese species of the genus *Psammoecus* Latreille, 1829 are taxonomically revised. Four new species, *P. scitus* **sp. n.** (misidentified with *P. quadrimaculatus*), *P. labyrinthicus* **sp. n.**, *P. boreas* **sp. n.** and *P. omotoensis* **sp. n.** are described. *Psammoecus bipunctatus* (Fabricius, 1792), *P. trimaculatus* Motschulsky, 1858 (misidentified with *P. triguttatus*), *P. simoni* Grouvelle, 1892, *P. fasciatus* Reitter, 1874 and *P. triguttatus* are redescribed. Another described species whose distribution in Japan is questionable. *P. quadrimaculatus* is also redescribed. Lectotype and paralectotype of *P. fasciatus* and *P. triguttatus* are designated.

## Introduction

The family Silvanidae Kirby 1837 (Coleoptera, Cucujoidea) includes two subfamilies and about 58 genera, approximately 500 species (Thomas and Leschen 2010) and is found around the world: 39 species have been recorded from Japan. The Silvanidae are considered to be fairly primitive among the Cucujoidea (Thomas 2002). Most members of the Silvanidae seem to be fungivorous, and share the character of a large pit for fungal transport called the “mycangium” on each mandible (Thomas 2002). However, Grebennikov and Leschen (2010) stated that these mycangial functions in the Silvanidae had not been verified experimentally. The Silvanidae contain some harmful pests of stored grains and grain products (e.g. *Ahasverus advena* (Waltl, 1834), *Oryzaephilus surinamensis* (Linnaeus, 1758) and *Silvanus lewisi* Reitter, 1876) (Halstead 1986). Some of these species are occasionally transferred with goods in transit and distributed over the world.

The genus *Psammoecus* Latreille, 1829 (Brontinae, Telephanini) includes about 80 described species and is the second largest genus in the Silvanidae (Thomas and Leschen 2010). Species belonging to *Psammoecus* were recorded only from the Old World for many years, but one species, *Psammoecus trimaculatus* Motschulsky, 1858 was found in Brazil recently (Thomas and Yamamoto 2007). In Japan, six described species and four undescribed species of *Psammoecus* were reported by Hirano (2009) and Hirano (2010). Pal (1985) carried out a historical review of *Psammoecus* in detail and concluded that *Psammoecus* belongs to the tribe Psammoecini of the subfamily Psammoecinae. Thomas and Nearns (2008) stated that the correct name of the tribe is Telephanini and that the Psammoecinae should be named Brontinae according to the principle of priority, and Karner (2012) followed this treatment. Lu and Han (2006) reported that *Psammoecus triguttatus* Reitter, 1874 had been transferred with leather and its packages in transit from China. Therefore, the species belonging to *Psammoecus* have a potential as important pests and alien species.

The species of *Psammoecus* are found in plant detritus and they are sometimes attracted to light (Karner 2012), but there is little information on their ecology. In addition, taxonomic studies of *Psammoecus* are also insufficient and confused, probably for the following reasons: 1) most *Psammoecus* species were described very early historically; 2) their coloration and the black maculae on the elytra are very variable (e.g. Kreissl 1976; Yoshida and Hirowatari 2013); 3) there are no more than three studies containing descriptions of the genital structures (Pal 1985; Karner 2012; Yoshida and Hirowatari 2013) which provide very useful characters for taxonomic study of the genus (Karner 2012); 4) their body sizes are relatively small, ranging from 1.8 mm to 3.6 mm. In Japan, the classification of *Psammoecus* has also been confused. For example, Hirano (2009) and Hirano (2010) reported four undescribed species from Japan and stated that the species illustrated as *Psammoecus triguttatus* by Nakane (1963) seemed to be an undescribed species. In addition, Hirano (2009) and Hirano (2010) suspected the authenticity of records of *Psammoecus trimaculatus* from Japan. Recently, Yoshida and Hirowatari (2013) added a new species, *Psammoecus hiranoi*, to the Japanese fauna.

As stated above, taxonomy of this genus involves many problems and it has a potential to provide important pests and alien species. Hence, the authors here review the classification of Japanese *Psammoecus* by comparison of morphological characters and seek to resolve the taxonomic problems.

## Materials and methods

### Observation of morphology and dissection and photographic technique

External characters were observed under a stereoscopic microscope (Olympus SZX10). Genital structures were placed on a cavity slide glass with 50% glycerol solution and observed with an optical microscope (Nikon Eclipse E400). The genitalia slide was prepared in the following steps: the removed abdomen was placed in a 200 µl PCR tube filled with 10% solution of potassium hydroxide (KOH) and kept in heated water for about seven minutes. After rinsing in 70% ethanol solution, the abdomen was dissected by cutting its side using fine insect pins. The genitalia were removed and transferred to a cavity slide glass with 50% glycerol solution for observation. After the observation, the genitalia and the abdomen were mounted in Euparal on cover glasses each glued to a piece of cardboard, and pinned with the specimens.

Photographs were taken with digital camera (Canon EOS 7D), and composite images were produced using automontage software Combine ZM. These images were retouched using Photoshop 6.0 (Adobe Systems Inc.)

### Terminology, abbreviations and specimen deposition

Technical terms of genital structures follow Lawrence et al. (2010) and Lawrence et al. (2011). Morphological abbreviations are as follows: BL – body length from anterior margin of clypeus to apex of elytra measured along the median line; PL – length of pronotum measured along the median line; PW – greatest width of pronotum including lateral teeth; EW – greatest width of elytra; HL – head length from base to anterior margin of clypeus; HW – head width across eyes; IE – width of distance between eyes.

Depositories of the examined specimens are in the Ehime University Museum, Matsuyama (EUMJ), the Natural History Museum, London (BMNH), the Osaka Museum of Natural History, Osaka (OMNH), the Systematic Entomology, Hokkaido University, Sapporo (SEHU), Isamu Tanaka Collection, Nishinomiya (ITC), Yukihiko Hirano Collection, Odawara (YHC) and the Entomological Laboratory, Kyushu University, Fukuoka (ELKU).

## Results and discussion

### Taxonomy

#### Psammoecus scitus sp. n.

http://zoobank.org/EEAFC47C-A1E6-4E6F-B4CF-0F099AE6686F

http://species-id.net/wiki/Psammoecus_scitus

Japanese name: Yotsumon-semaru-hiratamushi

**Figures 1.**
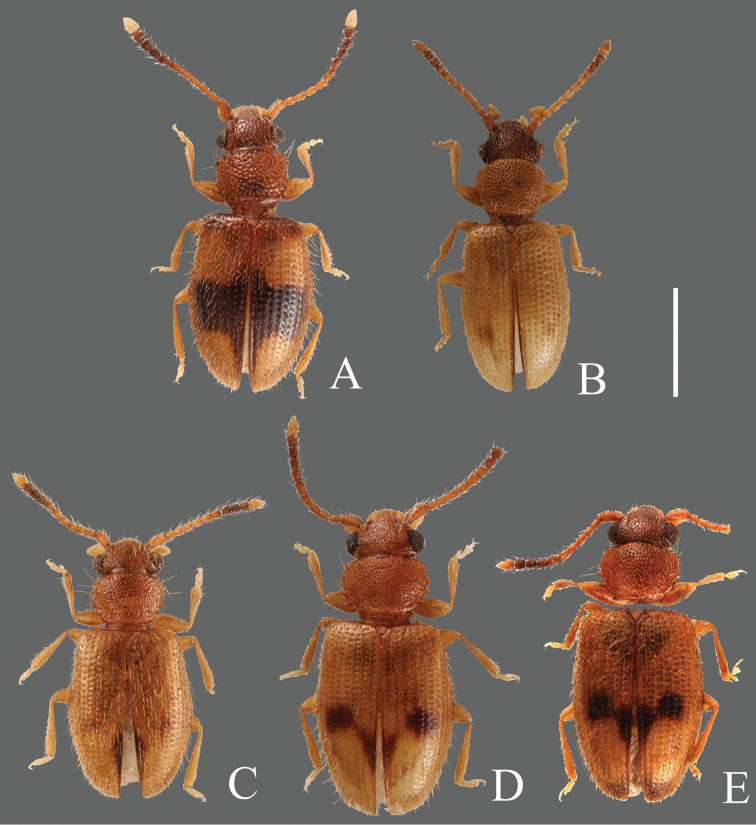
Habitus of *Psammoecus* spp. **A** *Psammoecus scitus* sp. n., holotype **B** *Psammoecus bipunctatus* (Fabricius, 1792) **C** *Psammoecus trimaculatus* Motschulsky, 1858 **D** *Psammoecus triguttatus* Reitter, 1874, lectotype **E** *Psammoecus labyrinthicus* sp. n., holotype. Scale: 1.0 mm.

Figs 1A , 3 and 13A–C

Psammoecus quadrimaculatus : Hirano 2009: 64, 65, 82, fig. 4. – Hirano 2010: 12, 14. (misidentification)

##### Diagnosis.

This species is similar to *Psammoecus hiranoi* Yoshida & Hirowatari, 2013, especially in darker specimens. However they can be distinguished by the ventral shape of the head. The temples of this species are immediately narrowed behind eyes, while those of *Psammoecus hiranoi* are widened behind the eyes and gradually narrowed toward the anterior margins.

##### Description.

**Body length.** 2.65–3.26 mm (n=48).

**Coloration** (Fig. 1A). Head and pronotum yellowish-brown to reddish-brown. Elytra black with yellowish-brown maculae; horizontal band at anterior 1/4, sometimes enlarged toward bases of elytra, round maculae at posterior 1/4, connected to lateral margins. Antennae yellowish-brown basally, 6th antennomere darker, 7th to 10th blackish-brown, 11th (apex) very bright.

**Head** (Fig. 3A, B, C). Broad, HW/HL 1.31–2.00; IE/HL 0.87–1.30. Temples immediately narrowed behind eyes, slightly incised at bases. Dorsal surface with comparatively coarse punctuation, ventral surface with very sparse punctures. Antennae 1.40–1.43 mm long, slender; covered relatively sparsely with long erect pubescence on each antennomere; approximate ratio of antennomeres of holotype as follows: 2.5: 1.1: 1.2: 1.3: 1.3: 1.3: 1.1: 1.2: 1.2: 1.0: 2.0 (Fig. 3A).

**Pronotum** (Figs 3B, C). Subquadrate, PW/PL 1.14–1.32. Punctuation on dorsal surface comparatively sparse, punctuation on ventral surface sparser than on dorsal surface. Pubescence composed of many short setae, and long setae on teeth on lateral margins and anterior and posterior angles. Anterior angles with a few very small protrusions, lateral margins with several small teeth, these teeth variable, slightly longer on lateral margins around anterior and posterior angles, and each posterior angle with very small teeth.

**Elytra** (Fig. 3E). Elongate-oval, EW/BL 0.38–0.51. Rows of punctures narrower than interstices. Pubescence composed of many semi-long medium length setae, and some long erect setae in a row around lateral margins, longer toward anterior portion.

**9th abdominal sternite** (Fig. 13A). Strut very long, narrow, cut at anterior 3/8, diverging gradually at posterior 1/8, branches comparatively small. Lateral sclerites elongate with small sclerites attached around posterior apical portion.

**Aedeagus** (Fig. 13B, C). Parameres broad, depressed at base, narrowed on inner margins in posterior half, incised around apex, punctuated very sparsely, a few long setae around apex, several short setae on lateral margins and posterior half of inner margins. Phallobase diverging in anterior half, each branch twisted at anterior 1/4. Penis relatively elongate, curved dorsally before apex.

##### Type series.

Holotype: male, Kûra, Ishigaki Island, Okinawa Prefecture, Japan, 22–IX–2012, T. Yoshida leg. (EUMJ). Paratypes: [Kagoshima Pref.] 1 ex., Honcha, Amami-Ôshima Island, 23–VII–1962, N. Ohbayashi leg. (EUMJ); 1 ex., Nadakawa, Amami-Ôshima Island, 16–VII–1962, N. Ohbayashi leg. (EUMJ); 1 ex., Kamiya, Amami-Ôshima Island, 4–XI–1984, M. Tomokuni leg. (EUMJ). [Okinawa Pref.] 1 ex., Hiji, Kunigami Village, Okinawa Island, 15–20–VI–1994, K. Okada leg. (EUMJ); 1 male & 1 female, Takazato, Kunigami Village, Okinawa Island, 18–22–VI–2003, I. Tanaka leg. (ITC); 2 exs., Inamine, Nago City, Okinawa Island, 26–IX–2012, T. Yoshida leg. (ELKU); 1 ex., Shokubutsuen, Miyako Island, 3–III–1999, T. Mizoguchi leg. (EUMJ); 1 female, Kûra, Ishigaki Island, 22–IX–2012, T. Yoshida leg. (ELKU); 4 exs., Ôura Dam, Ishigaki Island, 7–X–2013, R. Itô leg. (ELKU); 2 exs., Mt. Yarabu-dake, Ishigaki Island, 26–III–2000, T. Kurihara leg. (EUMJ); 7 exs., same locality, 16–VI–2002, T. Watanabe leg. (ELKU); 5 exs., same locality, 8–X–2013, R. Itô leg. (ELKU); 1 male, same locality, 8–III–2006, I. Tanaka leg. (ITC); 1 female, Kabira, Ishigaki Island, 19–IV–2010, I. Tanaka leg. (ITC); 2 exs., Takeda Rindô, Ishigaki Island, 14–III–1999, T. Mizoguchi leg. (EUMJ); 2 exs., same locality, 16–VI–2002, T. Watanabe leg. (ELKU); 2 exs., same locality, 22–IX–2012, T. Yoshida leg. (ELKU); 6 exs., same locality, 23–IX–2012, T. Yoshida leg. (ELKU); 3 exs., Funaura, Iriomote Island, 18–III–2008, S. Yamamoto leg. (ELKU); 1 ex., Urauchi-gawa, Iriomote Island, 2–VII–1994, K. Okada leg. (EUMJ); 3 exs., Komi, Iriomote Island, 20–III–2008, S. Yamamoto leg. (ELKU); 1 ex., Ôtomi Rindô, Iriomote Island, 13–III–1999, T. Mizoguchi leg. (EUMJ); 1 ex., Mt. Inbi-dake, Yonaguni Island, 31–III–2009, S. Yamamoto leg. (ELKU).

##### Distribution.

JAPAN: Amami-Ôshima, Okinawa, Miyako, Ishigaki, Iriomote and Yonaguni Islands.

##### Biological notes.

This species is found in piled dead leaves such as Japanese pampas grass *Miscanthus sinensis* Andersson, Banana *Musa* sp. and some other kinds of broad-leaved tree. This species is most common in the Nansei Islands.

##### Etymology.

The specific name means ‘pretty’ and ‘beautiful’. This new species has vivid coloration.

##### Remarks.

*Psammoecus scitus* sp. n. has been misidentified as *Psammoecus quadrimaculatus* Reitter, 1874 for a long time. *Psammoecus quadrimaculatus* was originally described from ‘Japonia’ (=Japan) by Reitter (1874). Sato (1989) first gave the Japanese name ‘Yotsumon-semaru-hiratamushi’ to *Psammoecus quadrimaculatus*. Hirano (2009) and Hirano (2010) provided figures of this species as *Psammoecus quadrimaculatus*. However, examination of the holotype of *Psammoecus quadrimaculatus* deposited in BMNH revealed that external characters such as the shape of the teeth on the lateral margins of the pronotum and the male genital structure are distinctly different.

#### Psammoecus bipunctatus

(Fabricius, 1792)

http://species-id.net/wiki/Psammoecus_bipunctatus

[Japanese name: Futamon-semaru-hiratamushi]

Figs 1B , 4 and 13D–F

Notoxus bipunctatus Fabricius, 1792: 212. Type locality: Germania Dom.
Latridius bipunctatus : Herbst 1793: 10.
Anthicus bipunctatus Fabricius, 1801: 291.
Psammoecus bipunctatus : Latreille 1829: 135. – Schaufuss 1916: 457, fig. 4 in pl. 14. – Hetschko 1930: 81. (catalogue) – Halstead et al. 2007 – Hirano 2009: 63, 64, 82, fig. 3. – Hirano 2010: 11, 12.
Psammaechus [sic.] *bipunctatus*: Smith 1851: 15. (catalogue)

##### Diagnosis.

This species is similar to *Psammoecus trimaculatus* and *Psammoecus boreas* sp. n., but can be distinguished from the former by the shorter teeth on the lateral margins of the pronotum and from the latter by the shorter body length and the shorter antennae.

##### Description.

**Body length.** 2.25–2.83 mm (n=9).

**Coloration** (Fig. 1B). Head dark brown, gradually darker toward anterior. Pronotum yellowish-brown to reddish-brown. Elytra yellowish-brown with round dark maculae on posterior 1/3, elytral suture dark in posterior half. Elytra of lighter colored specimens with reduced dark maculae and dark areas of elytral suture. Antennae yellowish-brown basally, 9th, 10th and posterior end of 8th antennomeres blackish-brown, 11th (apex) yellowish-brown.

**Head** (Fig. 4A, B, C). Triangular, HW/HL 1.55–2.06; IE/HL 1.03–1.33. Dorsal surface with strong dense punctures, temples and posterior ventral surface also with irregularly dense punctures. Temples well enlarged, immediately narrowing thereafter. Eyes prominent, relatively small. Antennae 1.09–1.20 mm, comparatively thick, short; covered with medium length erect pubescence on each antennomere; approximate ratio of one of examined specimens as follows: 2.6: 1.1: 1.2: 1.1: 1.2: 1.2: 1.1: 1.0: 1.1: 1.0: 1.8 (Fig. 4A).

**Pronotum** (Fig. 4B, C). Roundly subquadrate, PW/PL 1.16–1.24. Dorsal surface with strong moderately dense punctuation, denser than that on ventral surface; some punctures on ventral surface in rows. Pubescence composed of many short setae on dorsal surface and several medium length setae on each lateral margin, distance between medium length setae approximately regular. Each anterior angle with several very small teeth, each lateral margin with four similarly sized small teeth, the distance between them irregular and variable; each posterior angle with a small tooth, similar in size to those on lateral margins.

**Elytra** (Fig. 4E). Elongate, EW/BL 0.36–0.47. Rows of punctures almost same width as interstices. Pubescence composed of numerous comparatively short setae, without long setae.

**9th abdominal sternite** (Fig. 13D). Strut Y-shaped, cut at anterior 1/3, diverging in posterior 1/4, branches relatively wide, ends of each branch pointed and curved inwards. Lateral sclerites elongate, membranous.

**Aedeagus** (Figs 13E and F). Parameres club-shaped, wide and thick at bases, each apical portion with a long seta, stick-shaped portions with sparse punctuation and several short setae, lateral portions and inner margins of bases with successions of dense punctures, inner margin of bases with numerous short setae. Phallobase enlarged toward posterior margin from posterior 1/3, anterior margin roundly incised, anterior portion extended. Penis flat, wide, narrowed gradually toward apex, apical portion pointed, apical portion of dorsal part with sparse punctures.

##### Specimens examined.

JAPAN: [Hokkaido Pref.] 1 male, Takkobu-numa Lake, Kushiro City, 1–IX–2006, T. Yoshida leg. (ELKU); 1 ex., Iwabokki, Kushiro City, 25–VIII–1990, M. Sakai leg. (EUMJ); 6 exs., same locality, 26–VIII–1990, M. Sakai leg. (EUMJ); 1 ex., same locality, 29–VIII–1990, M. Sakai leg. (EUMJ).

##### Distribution.

JAPAN: Hokkaido; Africa; Europe; Russia; Turkestan.

##### Biological notes.

This species is known to inhabit marshland. Warne (1963) reported that large numbers of this species were found on *Carex*.

##### Remarks.

The habitat and distribution of this species are unusual in the genus *Psammoecus*. This species inhabits marshland and is distributed in comparatively high latitudes where species richness of *Psammoecus* is poor. Coloration is variable. In the past, four aberrations and two varieties (e.g. Lucas 1843; Gerhardt 1912) were described.

#### Psammoecus trimaculatus

Motschulsky, 1858

http://species-id.net/wiki/Psammoecus_trimaculatus

Japanese name: Mitsumon-semaru-hiratamushi

Figs 1C , 5 and 13G–I

Psammaechus [sic.] *trimaculatus* Motschulsky, 1858: *Etud. Ent.* 7: 45. Type locality: Sri Lanka; Type deposition: Zoological Museum, Moscow Lomonosov. (referring to Hetschko (1930))
Psammoecus trimaculatus : Waterhouse 1876: 124. – Reitter 1879: 509. – Grouvelle 1906: 125. – Grouvelle 1908: 476. – Hetschko 1930: 81. (catalogue) – Hisamatsu 1977: 21. – Hisamatsu 1982: 16. – Pal 1985: 41, Figs 15–29. – Sato 1989: 377. – Sasaki et al. 2002: 224. – Halstead et al. 2007 – Hirano 2009: 66. – Hirano 2010: 15. – Karner 2012: 24.
Psammoecus triguttatus : Hirano 2009: 64, 65, 66. – Hirano 2010: 12, 14. (misidentification)

##### Diagnosis.

This species is closely similar to *Psammoecus triguttatus* and *Psammoecus labyrinthicus* sp. n. but it can be distinguished from the former by the larger basal parameres and the broader distance between the posterior margin of the phallobase and the deepest point of incision of the anterior margin of the phallobase, and from the latter by the larger bases of parameres and the wider protuberances on the inner margins of the branches of the anterior phallobase. The rows of punctures on the elytra of *Psammoecus trimaculatus* (Fig. 5E) are comparatively narrower than in these two closely similar species. However, their external characters, especially their coloration, are variable. These features may cause misidentification.

##### Description.

**Body length.** 2.32–2.96 mm (n=38).

**9th abdominal sternite** (Fig. 13G). Strut Y-shaped, elongate, cut at anterior 3/8, diverging in posterior 1/5, branches relatively wide, narrowed gradually toward apex, ends of each branch curved inward. Lateral sclerites elongate, membranous.

**Aedeagus** (Fig. 13H, I). Parameres hatchet-shaped, inner margin around bases pointed, punctuated sparsely on stick-shaped portions, densely on anterior portions, a long seta at each apex, numerous short setae on stick-shaped portions and around inner margins of anterior parts. Posterior margin of phallobase rounded, phallobase roundly hollowed, its anterior branches markedly protruding inward. Penis gradually narrowed toward apex, posterior 1/9 flattened, punctuated sparsely on posterior 1/9.

##### Specimens examined.

India: [Andhra Pradesh State] 1 ex., Hyderabad, 18–X–1969, T. Ishihara leg. (EUMJ). JAPAN: [Hokkaido Pref.] 1 male, Suehiro, Asahikawa City, 18–IX–2013, T. Yoshida leg. (ELKU); 1 male, Aizankei, Kamikawa Town, 2–IX–1977, A. Oda leg. (EUMJ); 1 ex., Sôunkyo, Kamikawa Town, 4–IX–1977, A. Oda leg. (EUMJ); 1 ex., Chûbisei, Memuro Town, 25–VIII–1995, S. Hisamatsu leg. (EUMJ); 2 exs., Fushimi Marsh, Memuro Town, 25–VIII–1995, S. Hisamatsu leg. (EUMJ). [Aomori Pref.] 1 male, Namioka Ôaza Yoshinoda, Aomori City, 16–IX–2012, K. Ikeuchi leg. (ELKU). [Gifu Pref.] 1 male, Tentoku, Mizunami City, 31–VII–2009, K. Itô leg. (ELKU). [Hyogo Pref.] 1 male, Mt. Mikusa-yama, Inagawa Town, 27–VI–1991, K. Ikeuchi leg. (ELKU). [Wakayama Pref.] 2 males, 3 females & 7 exs., Biwadani, Kinokawa City, 25–IV–2012, T. Yoshida leg. (ELKU). [Kagawa Pref.] 1 male, Yoshima Island, Sakaide City, 10–12–IX–1973, S. Kinoshita leg. (EUMJ). [Tokushima Pref.] 2 males, 2 females & 4 exs., Kamojima-chô, Yoshinogawa City, 4–III–2012, T. Yoshida leg. (ELKU). [Kochi Pref.] 1 male, Godaisan-Park, Kochi City, 21–V–1983, K. Ishida leg. (EUMJ). [Fukuoka Pref.] 1 male, Kitano-chô, Kurume City, 1–VII–2013, T. Yoshida leg. (ELKU). [Kagoshima Pref.] 1 male, Kotoku-gawa, Amami-Ôshima Island, 14–III–1988, M. Satô leg. (EUMJ). [Okinawa Pref.] 1 male, Yona, Kunigami Village, Okinawa Island, 17–VII–1965, Y. Hori leg. (EUMJ); 1 male, same locality, 19–X–1987, M. Sakai leg. (EUMJ); 1 male & 1 ex., Inaba, Iriomote Island, 9–VIII–1962, M. Satô & Y. Arita leg. (EUMJ).

##### Distribution.

JAPAN: Hokkaido, Honshu, Shikoku, Kyushu, Amami-Ôshima, Okinawa, and Iriomote Islands. (Madagascar recorded by Karner (2012); Nepal, India and Bhutan recorded by Pal (1980).)

##### Remarks.

This species was redescribed by Pal (1980) with a description of the larva. It is distributed worldwide except for Europe and North America and common at least in Japan and India (Hirano 2009; Karner 2012; Pal 1980).

Hirano (2009) and Hirano (2010) illustrated this species as *Psammoecus triguttatus* and suggested that the distribution of *Psammoecus trimaculatus* in Japan is doubtful. In this paper, it is regarded as *Psammoecus trimaculatus* on the basis of comparison of the male genital structure of the species illustrated by Pal (1985).

Pal (1985) gave as the distribution of this species Madagascar, Sri Lanka, Nepal, India, Malaysia, Burma, New Guinea, Australia and Japan, synonymizing the following species with *Psammoecus trimaculatus*: *Telephanus cruciger* Waterhouse, 1876 and *Psammoecus cephalotes* Grouvelle, 1919 from New Guinea, *Cucujus incommodus* Walker, 1859 from Sri Lanka and *Psammoecus ypsilon* Blackburn, 1903 from Australia (Walker 1859; Waterhouse 1876; Blackburn 1903; Grouvelle 1919). In addition, Thomas and Yamamoto (2007) recorded this species from Brazil. However, in view of the evidence that at least two other closely similar species have been confused with this species, past records of this species should be reexamined. The specimen figured by Thomas and Yamamoto (2007) seems to be another species. In this paper, records of this species from outside Japan are recognised from label data described by Pal (1980) and Karner (2012), on the basis that they examined the male genital structure.

#### Psammoecus triguttatus

Reitter, 1874

http://species-id.net/wiki/Psammoecus_triguttatus

Japanese name: Nise-mitsumon-semaru-hiratamushi

Figs 1D , 6 and 13J–L

Psamoecus triguttatus [sic.] Reitter, 1874: 524. Type locality: Japan; Type deposition: the Natural History Museum, London; Type examined. (misspelling)
Psammoecus triguttata [sic.]: Hisamatsu 1982: 16.
Psammoecus triguttatus : Hetschko 1930: 82. (catalogue) – Kamiya 1961: 18, pl. 5. – Sasaji 1985: 204, fig. 33 in pl. 32. – Halstead et al. 2007

##### Diagnosis.

This species is closely similar to *Psammoecus trimaculatus* and *Psammoecus labyrinthicus* sp. n. Morphological differences among these species were stated in diagnosis of *Psammoecus trimaculatus*.

**Description. Body length.** 2.32–2.93 mm (n=23).

**Coloration** (Fig. 1D). Head and pronotum yellowish-brown to reddish-brown. Elytra yellowish-brown with variable dark maculae: round ones at half, oval ones on the posterior half to posterior 1/4 of elytral suture, sometimes connected with maculae at half, darkened around end of elytra. Antennae reddish-brown basally, blackish-brown from 8th to 10th antennomeres, 11th (apex) comparatively bright.

**Head** (Fig. 6A, B, C). Triangular, HW/HL 1.46–1.90; IE/HL 0.92–1.20. Temples slightly expanded behind eyes, narrowed toward posterior. Eyes large, prominent. Dorsal surface with relatively dense punctuation. Antennae 1.06–1.38 mm, moderately long; covered with considerable medium length or long semi-erect setae and short pubescence on each antennomere; approximate ratio of lectotype as follows: 2.7: 1.0: 1.1: 1.2: 1.3: 1.3: 1.3: 1.1: 1.1: 1.1: 2.0 (Fig. 6A).

**Pronotum** (Fig. 6B, C). Broad, PW/PL 1.31–1.50. Dorsal surface with relatively dense punctuation. Pubescence composed of relatively fine short or medium length setae, a long seta on each tooth on lateral margins and anterior and posterior angles. Each anterior angle with four or five small teeth; each lateral margin with four teeth, tooth I small, tooth II longer than tooth I, teeth III and IV relatively narrow and of almost identical size, longer than tooth II, tooth V smallest or absent; each posterior angle with a few very small protuberances of variable shape.

**Elytra** (Fig. 6E). Elongate-oval, EW/BL 0.39–0.45. Rows of punctures narrower than interstices. Pubescence composed of many semi-erect setae of medium length; several long setae in a row around anterolateral margins.

**9th abdominal sternite** (Fig. 13J). Strut Y-shaped, cut at anterior 2/5. Lateral sclerites curved inward, relatively elongate.

**Aedeagus** (Fig. 13K, L). Parameres club-shaped, wide around bases, posterior inner margins of wide portions a little prominent, punctuation on narrow portions relatively sparse, a little denser on wide portions, posterior half of inner margins of wide portions with many setae, narrow portions with several sparse setae, apex with a long seta. Posterior half of phallobase wider towards posterior margin, distance between posterior margin and deepest point of incision of margin of upper layer narrow, protuberances around anterior 1/4 pointed inwards, posterior margin of lower layer widely deeply incised. Penis relatively flat and thin, narrowed toward apex, punctuation around apex denser toward apex.

##### Type series.

Lectotype: male, Nagasaki, Nagasaki Prefecture, Japan, 1869, G. Lewis leg. (BMNH). Paralectotype: 1 female, same locality, 1869, G. Lewis leg. (BMNH). (here designated)

##### Specimens examined.

JAPAM: [Tokyo Pref.] 1 male, Hatagaya, Shibuya-ku, 30–VII–1956, K. Tanaka leg. (EUMJ). [Gifu Pref.] 1 male, Tentoku, Toki-chô, Mizunami City, 6–VIII–2010, K. Itô leg. (ELKU); 1 male, Konokure, Toki-chô, Mizunami City, 16–VII–2011, K. Itô leg. (ELKU). [Kagoshima Pref.] 1 male, 4 females and 3exs., Sata Cape, Minamiôsumi Town, 6–VII–1968, K. Suga leg. (HUSE); 1 male, Nakanoshima Island, Toshima Village, 9–VII–1969, M. Satô leg. (EUMJ); 1 male, same locality, 26–VI–1965, S. Fukuda leg. (HUSE). [Okinawa Pref.] 1 male, Aha beach, Kunigami Village, Okinawa Island, 23–I–2008, Y. Hirano leg. (YHC); 2 males and 2 exs., Motobu Town, Okinawa Island, 19–VIII–2008, T. Yoshida leg. (ELKU); 1 male, Fusato, Nanjô City, Okinawa Island, 5–VI–1970, M. Takagi leg. (EUMJ); 1 male, Mt. Omoto-san, Ishigaki Island, 1–VII–1965, Y. Hori leg. (EUMJ); 1 male, Ushuku-no-mori, Iriomote Island, 26–VI–1965, Y. Hori leg. (EUMJ).

##### Distribution.

JAPAN: Honshu, Kyushu, Nakanoshima (Tokara Islands), Okinawa, Ishigaki and Iriomote Islands.

##### Remarks.

*Psammoecus* sp. 4 illustrated by Hirano (2009) and Hirano (2010) seems to be this species or *Psammoecus labyrinthicus* sp. n. However, identification is difficult, because the specimen is a female. The Japanese name of *Psammoecus* sp. 4 given by Hirano (2009) and Hirano (2010) “Nise-mitsumon-semaru-hiratamushi” is adopted as the Japanese name of this species in this paper.

Halstead et al. (2007) recorded distribution of this species from Russia (Far East), Korea, China and Japan. However, it is now clear that at least two closely similar species occur in Japan, so, past records of this species should be reconfirmed. In addition, two or more closely similar species were sometimes found in the same limited area such as Tentoku, Toki-cho, Mizunami City, Gifu Prefecture where *Psammoecus triguttatus* and *Psammoecus trimaculatus* were collected. Thus, these species should be identified carefully.

Syntypes of this species consist of two specimens, one male and one female. The male specimen is designated as a lectotype, and the female specimen is designated as a paralectotype.

#### Psammoecus labyrinthicus sp. n.

http://zoobank.org/D11F2212-7A76-46A8-951C-B3FCC1543BCC

http://species-id.net/wiki/Psammoecus_labyrinthicus

[Japanese name: Hachijô-mitsumon-semaru-hiratamushi]

Figs 1E , 7 and 14A–C

##### Diagnosis.

This species is closely similar to *Psammoecus trimaculatus* and *Psammoecus triguttatus*. However, it can be distinguished by the male genital structure, especially the shape of the parameres, and the comparatively dense punctuation of the dorsal pronotum.

##### Description.

**Body length:** 2.66–3.38 mm (n=25).

**Coloration** (Fig. 1E). Head and pronotum reddish-brown. Elytra yellowish-brown with dark maculae; round ones at half, oblong ones on the posterior half to posterior 1/4 along elytral suture, darkened around end of elytra: these maculae sometimes connected each other. Antennae reddish-brown basally, 7th antennomere darkened, from 8th to 10th blackish-brown, 11th (apex) comparatively bright.

**Head** (Fig. 7A, B, C). Rounded-triangular, HW/HL 1.25–2.00; IE/HL 0.75–1.27. Temples slightly expanded behind eyes, gradually narrowed. Eyes large, prominent. Dorsal surface with dense and strong punctuation. Antennae 1.35–1.56 mm, moderately long; covered with medium length semi-erect pubescence and some long erect setae on each antennomere; approximate ratio of holotype as follows: 2.4: 1.0: 1.0: 1.2: 1.3: 1.1: 1.1: 1.1: 1.1: 1.1: 1.9 (Fig. 7A).

**Pronotum** (Fig. 7B, C). Roundly subquadrate, PW/PL 1.25–1.53. Relatively strong and dense punctuation on dorsal surface. Pubescence composed of short setae, a long seta on each tooth on lateral margins and anterior and posterior angles. Each anterior angle with several small teeth, each lateral margin with four short teeth; tooth I small, tooth II longer than tooth I, teeth III and IV almost the same size, longer than tooth II, teeth II, III and IV broadened at base, each posterior angle with a very small tooth, the shape of these teeth variable.

**Elytra** (Fig. 7E). Elongate-oval, EW/BL 0.41–0.47. Rows of punctures narrower than interstices. Pubescence composed of numerous medium length semi-erect setae and some long setae in a row around lateral margins.

**9th abdominal sternite** (Fig. 14A). Strut relatively short, cut at anterior 1/3. Lateral sclerites fused with strut, curved inwards.

**Aedeagus** (Fig. 14B, C). Parameres stout, club-shaped, punctuated on anterior half of inner margins, anterolateral portions well punctuated, anterior half of inner margins with many setae of variable size, apex with a long seta. Phallobase broadened toward posterior margin, posterior margin widely incised, protuberances around 1/3 of inner margins of branches, especially right protuberance thin. Penis flat, narrowed toward apex, apex pointed, punctuated around anterior 1/8, apex densely punctuated.

##### Type series.

Holotype: male, Hachijô Island, Hachijô Town, Tokyo Prefecture, Japan, 21–VII–1957, S. Hisamatsu leg. (EUMJ). Paratypes: [Tokyo Pref.] 3 females, same data as holotype. (EUMJ). [Mie Pref.] 4 males, Ujitachi-chô, Ise City, 29–VIII–2009, N. Narukawa leg. (ELKU). [Nagasaki Pref.] 1 male and 11 exs., Ta, Toyotama-chô, Tsushima Island, 7–VII–2013, T. Yoshida leg. (ELKU). [Kagoshima Pref.] 2 males and 3 exs., Takarajima Island, Toshima Village, 2–VII–1960, M. Satô leg. (EUMJ); 1 ex., same locality, 3–VII–1960, M. Satô leg. (EUMJ); 1 ex., same locality, 1–VI–1962, M. Satô leg. (EUMJ).

##### Distribution.

JAPAN: Honshu, Hachijô (Izu Islands), Tsushima, Takarajima Islands (Tokara Islands).

##### Biological notes.

The first author (Yoshida) collected many individuals of this new species from dead leaves of evergreen trees in Tsushima Island, Nagasaki Prefecture (Fig. 15A).

##### Etymology.

The specific name means ‘labyrinthine’. Identification of *Psammoecus trimaculatus* and closely similar species is very difficult. The addition of this new species to this group of closely similar species made their identification more difficult like the labyrinth.

##### Remarks.

*Psammoecus trimaculatus* is closely related to this species, however the former has been often collected from dead leaves of monocotyledon plant. These two species may have each distinct ecological trait.

#### Psammoecus boreas sp. n.

http://zoobank.org/1E58DE65-FC6D-4E40-B55E-A952B311A454

http://species-id.net/wiki/Psammoecus_boreas

Japanese name: Arame-semaru-hiratamushi

**Figures 2.**
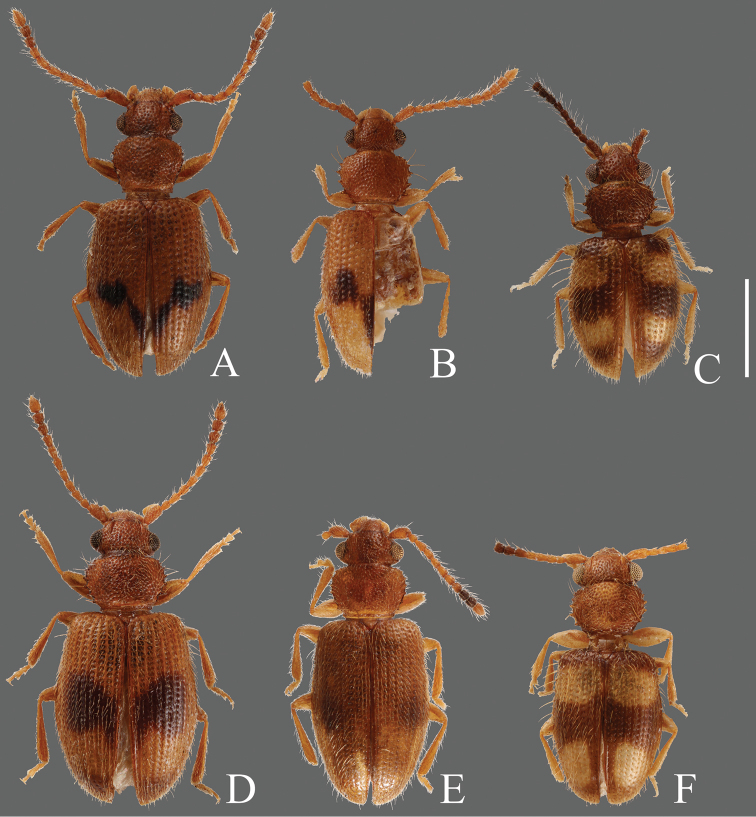
Habitus of *Psammoecus* spp. **A** *Psammoecus boreas* sp. n., holotype **B** *Psammoecus omotoensis* sp. n., holotype **C** *Psammoecus simoni* Grouvelle, 1892 **D** *Psammoecus fasciatus* Reitter, 1874, lectotype **E** *Psammoecus hiranoi* Yoshida & Hirowatari, 2013 **F** *Psammoecus quadrimaculatus* Reitter, 1874, holotype. Scale: 1.0 mm.

Figs 2A , 8 and 14D–F

Psammoecus triguttatus : Nakane 1963: 195, fig. 16 in pl. 98.
Psammoecus sp. 3, Hirano 2009: 63, 66, 67, fig. 8. – Hirano 2010: 12, 16.

##### Diagnosis.

This species is similar to *Psammoecus trimaculatus* and other species closely similar to *Psammoecus trimaculatus*. Nakane (1963) provided a figure of this species as *Psammoecus triguttatus*. It differs from the aforementioned species by the shorter lateral teeth of the pronotum. It is also very similar to *Psammoecus harmandi* Grouvelle, 1912 both in external characters and male genital structure as illustrated by Pal (1980), but can be distinguished from it by the longer antennae and the comparatively oblong 10th antennomere.

##### Description.

**Body length.** 2.74–3.27 mm (n=19).

**Coloration** (Fig. 2A). Head and pronotum yellowish-brown. Elytra yellowish-brown with dark brown maculae at half, oval ones at center of each elytron and dark oblique bands toward posterior portion, sometimes connected at elytral suture, forming a V-shaped band. Elytra of lighter color specimens with reduced maculae, oval ones and bands separated. Antennae yellowish-brown basally, posterior ends of 8th to 10th antennomeres darkened, or all antennomeres yellowish-brown in lighter colored specimens.

**Head** (Fig. 8A, B, C). Broad, HW/HL 1.35–1.65; IE/HL 0.96–1.13. Temples narrowed around each base. Eyes small, moderately rounded. Dorsal surface with moderately dense punctuation, ventral surface punctuated sparsely. Antennae 1.54–1.71 mm, thin, very long; covered with considerable long semi-erect pubescence on each antennomere; approximate ratio of holotype as follows: 2.6: 1.0: 1.1: 1.2: 1.2: 1.3: 1.2: 1.0: 1.0: 1.1: 1.7 (Fig. 8A).

**Pronotum** (Fig. 8B, C). Subquadrate, PW/PL 1.18–1.30. Dorsolateral portions lightly impressed. Dorsal surface with relatively dense punctuation, no punctures around posterior margin, comparatively sparse punctuation on ventral surface. Pubescence composed of many short setae and fine long setae on teeth on lateral margins and anterior and posterior angles. Each anterior angle with a distinct group of a few very small teeth, each lateral margin with four small teeth of almost the same size, each posterior angle with a small tooth, almost the same size as those on lateral margins.

**Elytra** (Fig. 8E). Oval, EW/BL 0.32–0.45. Rows of punctures wider than interstices. Pubescence composed of many short setae, no long setae.

**9th abdominal sternite** (Fig. 14D). Strut Y-shaped, cut deeply at anterior 1/5, diverging for posterior 1/4. Lateral sclerites elongate.

**Aedeagus** (Fig. 14E, F). Parameres cone-shaped with almost even sparse punctuation, sparser on bases, a few long setae around apical portions, a few short setae distributed sparsely. Phallobase tube-like, consisting of two layers, anterior margin rounded, dorsal surface around anterior margin thin, protuberances of upper layer directed towards anterior portion, small protuberances at beginning of divergence of upper layer. Penis stout, punctuated on posterior 1/9, rather coarsely on ventral surface.

##### Type series.

Holotype: male, Yoshin, Tanzawa, Kanagawa Prefecture, Japan, 26–V–1989, Y. Hirano leg. (EUMJ). Paratypes: [Hokkaido Pref.] 2 exs., Chûbisei, Memuro Town, 25–VIII–1995, S. Hisamatsu leg. (EUMJ); 1 ex., Mt. Sapporo-dake, Sapporo City, 5–VIII–1970, S. Kinoshita leg. (EUMJ). [Niigata Pref.] 1 ex., Mikuni Touge, 1–VII–1967, K. Baba leg. (HUSE). [Kanagawa Pref.] 1 ex., Yoshin, Tanzawa, 26–V–1989, Y. Hirano leg. (YHC). [Nagano Pref.] 1 ex., Tokugo Touge, 29–VII–1955, T. Nakane leg. (HUSE); 5 exs., Ôbora, Ueda City, 24–VII–2013, T. Yoshida leg. (ELKU). [Oita Pref.] 6 ex., Mt. Sobo-san, 7–VI–2009, S. Yamamoto leg. (ELKU).

##### Distribution.

JAPAN: Hokkaido, Honshu, Kyushu.

##### Etymology.

The specific name is from the god of the north wind of ancient Greek mythology. Most *Psammoecus* species are distributed in tropical or subtropical zones, however, this new species is exceptionally distributed in Hokkaido or on mountains of high altitude located in Honshu and Kyushu.

##### Remarks.

*Psammoecus* sp. 3 illustrated by Hirano (2009) and Hirano (2010) was conspecific with this species and named same Japanese name proposed by him.

#### Psammoecus omotoensis sp. n.

http://zoobank.org/E64E6D2D-B71B-40FC-83FA-551947595432

http://species-id.net/wiki/Psammoecus_omotoensis

Japanese name: Higenaga-semaru-hiratamushi

Figs 2B , 9 and 14G–I

##### Diagnosis.

This species is similar to *Psammoecus trimaculatus* and other species closely similar to *Psammoecus trimaculatus*. However, it can be distinguished from them by the male genital structure, especially the parameres which are fused to the phallobase. The distinguishing external characters of this species are tooth IV of the lateral margins of pronotum extended in a posterolateral direction and the long antennae, especially the 1st antennomere.

##### Description.

**Body length.** 2.71–2.77 mm (n=2).

**Coloration** (Fig. 2B). Head and pronotum yellowish-brown to reddish-brown. Elytra yellowish-brown with dark maculae, round ones at half of each elytron, black ones on posterior half of elytral suture narrower toward posterior elytral suture, a thin short black band between these maculae. Antennae yellowish-brown, almost unicolorous, 11th (apical) antennomere comparatively bright.

**Head** (Fig. 9A, B, C). Transverse, HW/HL 1.59–1.63; IE/HL 1.07. Temples well enlarged behind eyes, narrowed at bases. Eyes small, moderately rounded. Frequent pubescence and very sparse punctuation on ventral surface, moderately dense punctuation on dorsal surface. Antennae 1.44–1.49 mm long; covered with frequent medium length semi-erect fine setae and some long erect setae on each antennomere; approximate ratio of holotype as follows: 2.6: 1.0: 1.2: 1.4: 1.4: 1.4: 1.3: 1.1: 1.2: 1.0: 1.9 (Fig. 9A).

**Pronotum** (Fig. 9B, C). Transverse, PW/PL 1.30–1.35. Punctuation on dorsal surface coarse. Pubescence composed of short fine setae, a long seta on each tooth on lateral margins and anterior and posterior angles. Each anterior angle with several very small prominences and two small teeth, each lateral margin with four teeth; tooth I comparatively small, teeth II and III almost the same size, longer than tooth I, tooth IV extended in posterolateral direction, longer than tooth II and III, each posterior angle with a very small protuberance.

**Elytra** (Fig. 9E). Oval, EW/BL 0.37–0.44. Around half of lateral margins relatively expanded. Rows of punctures wider than interstices. Pubescence composed of medium length semi-erect setae and a few long setae around anterolateral margins.

**9th abdominal sternite** (Fig. 14G) Strut Y-shaped, anterior 2/7 slightly thickened, cut from anterior 2/7, diverging in posterior 1/3, branches long, ends of each branch curved inwards. Lateral sclerites elongate, membranous.

**Aedeagus** (Figs 14H and I) Parameres fused with phallobase, incised shallowly at inner and lateral margins of bases, relatively stout, gradually curved inwards, three long setae around posterior 1/3, some short setae on posterior half, well punctuated on anterior half of inner margins. Phallobase broad and somewhat flat, anterior margin widely but shallowly incised, lateral margins slightly depressed around anterior 1/4. Penis wide, thinner toward apex, narrowed around apex, punctuated around apex, densely punctuated at apex.

##### Type series.

Holotype: male, Mt. Omoto-san, Ishigaki Island, Okinawa Prefecture, Japan, 23–III–2000, T. Kurihara leg. (EUMJ). Paratype: [Okinawa Pref.] 1 male, same data as holotype, T. Kurihara leg. (EUMJ).

##### Distribution.

JAPAN: Ishigaki Island.

##### Etymology.

The specific name is derived from the type locality of the new species, Mt. omoto-san, Ishigaki Island, Okinawa Prefecture.

#### Psammoecus simoni

Grouvelle, 1892

http://species-id.net/wiki/Psammoecus_simoni

Japanese name: Hababiro-semaru-hiratamushi

Figs 2C , 10 and 14J–L

Psammoecus simonis [sic.] Grouvelle, 1892: 287. Type locality: Philippine; Type deposition: Museum National d’Histoire Naturelle, Paris. (misspelling)
Psammoecus simoni : Grouvelle 1908: 476, 488. – Hetschko 1930: 81. (catalogue) – Pal 1985: 31, fig. 11. – Hirano 2009: 63, 65, 82, fig. 5. – Hirano 2010: 11, 14. – Karner 2012: 25, fig. 11.

##### Diagnosis.

This species is distinguished from other Japanese *Psammoecus* species by the black elytra with yellow maculae and the posterior teeth on lateral margins of pronotum longer than those of anterior margins.

##### Description.

**Body length.** 2.24–2.56 mm (n=8).

**9th abdominal sternite** (Fig. 14J). Strut comparatively short, cut at anterior 2/5, diverging gradually, branches comparatively long and wide. Lateral sclerites elongate, slightly curved inwards, apical sclerites pointed.

**Aedeagus** (Fig. 14K, L). Parameres fused with phallobase, wide, short, posterior and lateral portions punctuated sparsely, densely punctuated triangular areas in anterior portions, a few long setae and short setae on posterior half, several short folds around beginning of divergence of parameres, a few lines on upper densely punctuated areas. Phallobase stout, broad in lateral aspect, anterior margin straight, a few subparallel lines on posterior dorsal portion. Penis comparatively broad, flattened around apex, apical margin rounded, punctuated sparsely on apex.

##### Specimens examined.

JAPAN: [Okinawa Pref.] 1 male, 4 females & 2 exs., Inamine, Nago City, Okinawa Island, 26–IX–2012, T. Yoshida leg. (ELKU); 1 ex., Mt. Yarabu-dake, Ishigaki Island, 16–VI–2002, T. Watanabe leg. (ELKU).

##### Distribution.

JAPAN: Okinawa, Hateruma and Ishigaki Islands; Madagascar; India; Sri Lanka; Malaysia; Indonesia; Philippines.

##### Biological notes.

This species is found in dead leaves to which fungi are attached and occurs sympatrically with other Silvanid species such as *Psammoecus trimaculatus*, *Psammoecus scitus* sp. n., *Cryptamorpha desjardinsi* (Guérin-Méneville, 1844) and *Monanus concinnulus* (Walker, 1858) (Fig. 15B).

##### Remarks.

Pal (1980) and Karner (2012) redescribed this species. Grouvelle (1892) described it as ‘simonis’. However, Grouvelle (1908) referred to it with the specific name ‘simoni’. Pal (1980), Hirano (2010) and Karner (2012) used the latter spelling, which we adopt in the present study.

#### Psammoecus fasciatus

Reitter, 1874

http://species-id.net/wiki/Psammoecus_fasciatus

Japanese name: Kuroobi-semaru-hiratamushi

Figs 2D , 11 and 14M–O

Psamoecus [sic.] *fasciatus* Reitter, 1874: 525. Type locality: Japan; Type deposition: the Natural History Museum, London; Type examined. (misspelling)
Psammoecus fasciatus : Hetschko 1930: 82. (catalogue) – Nakane 1963: 196, fig. 16 in pl. 98. – Hisamatsu 1977: 21. – Sasaji 1985: 204, fig. 34 in pl. 32. – Sato 1989: 377. – Halstead et al. 2007 – Hirano 2009: 64. – Hirano 2010: 12, 13. – Yoshida and Hirowatari 2013: 5, 11, 16, 17.

##### Diagnosis.

This species is closely similar to *Psammoecus hiranoi*. It can be distinguished by the longer parameres, the shallower incision of the phallobase on the anterior 1/4 and the shorter teeth on the lateral margins of the pronotum.

##### Description.

**Body length.** 2.75–3.50 mm (n=49).

**Coloration** (Fig. 2D). Head and pronotum yellowish-brown to reddish-brown. Elytra with a variable horizontal dark band at middle, this band in lighter color specimens thinner towards elytral suture, lateral margins, apical portion and posterior elytral suture dark. Antennae brown or reddish-brown basally, 7th and 8th antennomeres darker, 9th and 10th almost black, 11th (apex) lighter than basal ones. Antennae of some specimens almost unicolorous.

**Head** (Fig. 11A, B, C). Broad, HW/HL 1.40–2.08; IE/HL 0.94–1.38. Temples enlarged behind eyes, narrowed gradually toward posterior margin. Eyes large, slightly prominent. Ventral surface with rough irregular punctuation, sparser punctuation on dorsal surface. Antennae 1.38–1.57 mm, 2nd antennomere short; covered with thick medium length pubescence and some long erect pubescence on each antennomere; approximate ratio of lectotype as follows: 2.5: 1.0: 1.3: 1.3: 1.3: 1.3: 1.3: 1.3: 1.3: 1.2: 1.8 (Fig. 11A).

**Pronotum** (Fig. 11B, C). Broad, PW/PL 0.69–1.52. Punctuation on dorsal surface very dense, but no punctures around posterior margin, punctuation on ventral surface sparser than dorsal surface, some of them in rows especially on posterior half. Pubescence composed of frequent short setae on dorsal surface and a long seta on each tooth on lateral margins and at anterior and posterior angles. Each anterior angle with a distinct group of a few very small teeth, each lateral margin with four teeth; tooth I comparatively small, teeth II, III and IV almost the same moderate size, each posterior angle with a comparatively small tooth.

**Elytra** (Fig. 11E). Elongate-oval, EW/BL 0.41–0.47. Punctures wider than interstices. Pubescence composed of semi-erect medium length setae and some long erect setae in a row around lateral margins, longer toward anterior portion.

**9th abdominal sternite** (Fig. 14M). Strut cut at around half, diverging widely around the posterior base, ends of each branch curved inwards. Lateral sclerites triangular, flat.

**Aedeagus** (Figs 14N and O). Parameres simple stick-shaped, elongate, slightly curved inwards, punctuated sparsely except base, a few semi-long setae around apical portion. Phallobase diverging gradually around anterior 1/4, a little bulging along inner margins of each branch, lateral margins broadening. Penis also elongate, punctuated on posterior 1/6, more densely on ventral portion, especially densely on apical portion.

##### Type series.

Lectotype: male, Mt. Maya-san, Kobe City, Hyogo Prefecture, Japan, 1871, G. Lewis leg. (BMNH). Paralectotype: 1 female, same locality, 1871, G. Lewis leg. (BMNH). (here designated)

##### Specimens examined.

Russia: [Primorsky Krai] 1 ex., Chuguevka Village, 27–VI–1999, Y. Notsu leg. (EUMJ). JAPAN: [Niigata Pref.] 1 male & 1 ex., Renge-Onsen, Itoigawa City, 14–VIII–2003, I. Tanaka leg. (ITC). [Tokyo Pref.] 7 exs., Mikura Island, Mikurajima Village, 11–VI–1977, T. Nakane leg. (HUSE). [Nagano Pref.] 1 ex., Togakushi-Kôgen, Nagano City, 5–6–VII–2008, K. Mizuno leg. (KMC); 2 ex., Ôbora, Ueda City, 24–VII–2013, T. Yoshida leg. (ELKU). [Kyoto Pref.] 1 ex., Kifune, Kyoto City, 6–IV–1988, I. Tanaka leg. (ITC). [Hyogo Pref.] 1 ex., Itoi, Asago City, 26–IX–2004, I. Tanaka leg. (ITC); 5 exs., Sakanotani, Shisô City, 19–VII–2009, K. Itô leg. (KMC); 1 ex., Onzui, Shisô City, 19–VII–2009, K. Utô leg. (KMC); 1 ex., Mt. Yuzuruha-san, Nantan Town, Awaji Island, 18–X–2003, I. Tanaka leg. (ITC). [Nara Pref.] 1 ex., Mt. Kasuga-yama, Nara City, 5–V–1985, I. Tanaka leg. (ITC); 2 exs., same locality, 5–V–2012, K. Ikeuchi leg. (ELKU); 1 ex., same locality, 31–VII–2012, K. Matsuda leg. (ELKU). [Tokushima Pref.] 1 ex., Mt. Shibakoya-yama, Kamiyama Town, 31–VII–1975, M. Yoshida leg. (EUMJ). [Ehime Pref.] 1 ex., Nishidani, Yanadani Village, 15–16–VII–1994, K. Aita leg. (EUMJ). [Kochi Pref.] 1 ex., Muroto Cape, Muroto City, 7–VI–1959, S. Hisamatsu leg. (EUMJ). [Nagasaki Pref.] 1 ex., Mt. Tatera-san, Tsushima Island 16–IX–1995, N. Narukawa leg. (ELKU); 3 exs., Midake, 12–X–1977, Y. Notsu leg. (EUMJ). [Kagoshima Pref.] 1 male & 4 exs., Kirishima, 29–VII–1971, T. Nakane leg. (HUSE); 2 exs., Shiratani-unsuikyô, Yakushima Island, 15–VIII–2005, J. Ogawa leg. (EUMJ); 1 male & 1 ex., Onoaida Hodô, Yakushima Island, 14–15–IX–2002, N. Ohbayashi leg. (EUMJ); 2 exs., Kosugidani, Yakushima Island, 2–V–1984, K. Mizuno leg. (OMNH); 4 exs., Anbô, Yakushima Island, 16–VII–1989, N. Narukawa leg. (ELKU); 5 exs., Nogi, Nishino-omote, Tanegashima Island, 10–VII–1974, S. Hisamatsu leg. (EUMJ).

##### Distribution.

JAPAN: Hokkaido, Honshu, Shikoku, Kyushu, Mikura (Izu Islands), Awaji, Yakushima and Tanegashima Islands; Burma; Korea; Russia.

##### Biological notes.

The first author (Yoshida) collected this species from various kinds of dead leaves from evergreen trees, deciduous trees and bamboo.

##### Remarks.

The syntypes of this species consist of three specimens: two of them belong to this species, but the remaining one represents *Psammoecus trimaculatus* or *Psammoecus triguttatus*. We designate a male specimen as lectotype, and a female specimen as paralectotype. The type specimens were collected from Mt. Maya-san, Kobe City, Hyogo.

#### Psammoecus hiranoi

Yoshida & Hirowatari, 2013

http://species-id.net/wiki/Psammoecus_hiranoi

Japanese name: Herimon-semaru-hiratamushi

Fig. 2E

Psammoecus sp. 1: Hirano 2009: 63, 66, 82, fig. 6. – Hirano 2010: 12, 15.
Psammoecus sp. 2: Hirano 2009: 63, 66, 82, fig. 7. – Hirano 2010: 12, 15.
Psammoecus hiranoi Yoshida and Hirowatari 2013: 86–90.

##### Diagnosis.

This species is closely similar to *Psammoecus fasciatus* and morphological differences between these two species were stated in diagnosis of *Psammoecus fasciatus*.

##### Remarks.

Yoshida and Hirowatari (2013) described this species from the Nansei Islands, Japan including Nakanoshima (Tokara Islands), Amami-Ôshima, Tokunoshima, Okinawa, Ishigaki and the Iriomote Islands. It is closely similar to *Psammoecus fasciatus*, and these two species occur allopatrically across the Watase Line, which is one of the biogeographic borders proposed between the Palaearctic and Oriental regions passing through the Tokara Straits (Yoshida and Hirowatari 2013).

Yoshida and Hirowatari (2013) described this species and regarded the undetermined species, *Psammoecus* sp. 2 of Hirano (2009) and Hirano (2010), as conspecific. In addition, in the present study, *Psammoecus* sp. 1 illustrated by Hirano (2009) and Hirano (2010), which was represented by only one female, is also found to be conspecific with this species. We had the opportunity to examine some specimens possessing features of *Psammoecus* sp. 1 (Fig. 2E) and were able to conclude that morphological characters including the male genital structure of these specimens belonged to the range of morphological variation of *Psammoecus hiranoi*.

The following specimens were found after description of this species:

##### Specimens examined.

JAPAN: [Okinawa Pref.] 1 male & 4 exs., Mt. Yonaha-dake, Kunigami Village, Okinawa Island, 3–IV–1974, T. Kinoshita leg. (EUMJ); 10 exs., Ôkuni-rindô, Kuigami Village, Okinawa Island, 4–XI–2013, T. Yoshida leg. (ELKU).

#### Psammoecus quadrimaculatus

Reitter, 1874

http://species-id.net/wiki/Psammoecus_quadrimaculatus

Japanese name: Ruisu-yotsumon-semaru-hiratamushi

Figs 2F , 12 and 14P–R

Psamoecus [sic.] *quadrimaculatus* Reitter, 1874: 525. Type locality: Japan; Type deposition: the Natural History Museum, London; Type examined. (misspelling)

##### Diagnosis.

This species is similar to *Psammoecus trimaculatus*, *Psammoecus triguttatus* and *Psammoecus labyrinthicus* sp. n., but can be distinguished from *Psammoecus trimaculatus* and *Psammoecus triguttatus* by the wide triangular basal portion of the parameres and the apically narrow portion of the penis, and from *Psammoecus labyrinthicus* sp. n. by the longer parameres, the apically narrow portion of the penis and the shape of the phallobase.

##### Description.

**Body length.** 2.50 mm (n=1).

**Coloration** (Fig. 2F). Head reddish-brown, pronotum somewhat light reddish-brown. Elytra blackish-brown with four large yellowish-brown maculae; macula around anterior 1/4 of each elytron almost quadrate, macula on posterior half longer than wide. Antennae yellowish-brown basally, 6th to 10th antennomeres black, 6th slightly brighter, 11th (apex) bright.

**Head** (Figs 12A, B and C). Rounded-triangular, HW/HL 1.68; IE/HL 1.09. Temples slightly expanded behind eyes, narrowed at base. Eyes large, prominent, diameter about half of length of head. Punctuation of dorsal surface moderately dense, on ventral surface sparse, and absent on center portion of ventral surface. Antennae 1.44 mm; covered with medium length pubescence and some relatively long erect setae on each antennomere; approximate ratio of holotype as follows: 2.4: 1.0: 1.0: 1.1: 1.1: 1.3: 1.0: 1.1: 1.2: 1.1: 2.2 (Fig. 12A).

**Pronotum** (Fig. 12B, C). Roundly subquadrate, PW/PL 1.34. Punctuation on dorsal surface relatively strong and moderately sparse. Pubescence composed of medium length setae, a long seta on each tooth on lateral margins and anterior angles, a relatively long seta on each posterior angle. Each anterior angle with several small teeth, each lateral margin with four short teeth; tooth I small, tooth II longer than tooth I, teeth III and IV almost same size, longer than tooth II, teeth II, III and IV relatively widened around base, each posterior angle with a few very small teeth.

**Elytra** (Fig. 12E). Elongate-oval, EW/BL 0.46. Rows of punctures wider than interstices. Pubescence composed of many medium length semi-erect setae, long erect setae in a row on lateral margins.

**9th abdominal sternite** (Fig. 14P). Strut cut at anterior 1/3, diverging deeply around posterior 1/3. Lateral sclerites rhomboid, comparatively large, curved inwardly.

**Aedeagus** (Fig. 14Q, R). Parameres club-shaped; narrow portions relatively broad, punctuated sparsely, with several sparse setae, apex with a long seta; wide portions punctuated densely on posterior half of inner margins and anterolateral portions of outer margins, posterior half of inner margins with many setae. Phallobase consisting of two layers, posterior margin incised roundly, distance between posterior margin and deepest point of incision of margin of upper layer narrow, anterior margin of lower layer relatively narrowly incised, protuberances around anterior 1/4 narrow, projecting inwards, posterior margin of lower layer broadly incised. Penis relatively elongate and flat, with relatively dense punctuation on posterior 1/8.

##### Type series.

Holotype: male, Nagasaki, Nagasaki Prefecture, Japan, 1869, G. Lewis leg. (BMNH).

##### Distribution.

JAPAN: Nagasaki?.

##### Remarks.

Type specimen was mounted with a label reading ‘Nagasaki | 1869 | ? imported in Rice –’. We have not been able to find any specimen of this species from Japan other than holotype. Hence, occurrence of this species in Japan seems to be questionable.

### Key to species of the genus *Psammoecus* of Japan

**Table:** 

1	Teeth of lateral margins of pronotum relatively short and of identical size	2
–	Teeth of lateral margins of pronotum relatively long. Posterior teeth longer than those of anterior margins	6
2	1st antennomere longer than combined length of 2nd and 3rd antennomeres. Dorsolateral portions of pronotum impressed lightly	*Psammoecus boreas* sp. n.
–	1st antennomere almost as long as or shorter than combined length of 2nd and 3rd antennomeres. Dorsolateral portions of pronotum with no impressions	3
3	No long seta on anterolateral margins of elytra. Body oblong	*Psammoecus bipunctatus* (Fabricius, 1792)
–	Some long setae on anterolateral margins of elytra	4
4	Distance between teeth of lateral margins of pronotum irregular	*Psammoecus scitus* sp. n.
–	Distance between teeth of lateral margins of pronotum regular	5
5	Parameres long. Incision of anterior margin shallow	*Psammoecus fasciatus* Reitter, 1874
–	Parameres short. Incision of anterior margin deep	*Psammoecus hiranoi* Yoshida & Hirowatari, 2013
6	Black elytra with yellow maculae	*Psammoecus simoni* Grouvelle, 1892
–	Yellow elytra with black maculae or no maculae	7
7	Parameres fused with phallobase. Tooth IV of lateral margins of pronotum extended in a posterolateral direction. Antennae, especially 1st antennomere, long	*Psammoecus omotoensis* sp. n.
–	Parameres and phallobase divided. Tooth IV of lateral margins of pronotum extended in a roughly lateral direction. Antennae moderately long	8
8	Basal parameres large. Distance between posterior margin of phallobase and deepest point of incision of anterior margin broad. Rows of punctures on elytra comparatively narrow	*Psammoecus trimaculatus* Motschulsky, 1858
–	Basal parameres comparatively narrow. Rows of punctures on elytra comparatively wide	9
9	Distance between posterior margin of phallobase and deepest point of incision of anterior margin broad. Wide basal portion of parameres square. Punctuation of pronotal disk moderately dense	*Psammoecus labyrinthicus* sp. n.
–	Distance between posterior margin of phallobase and deepest point of incision of anterior margin narrow. Wide basal portion of parameres triangular. Punctuation of pronotal disk comparatively dense	*Psammoecus triguttatus* Reitter, 1874

**Figures 3.**
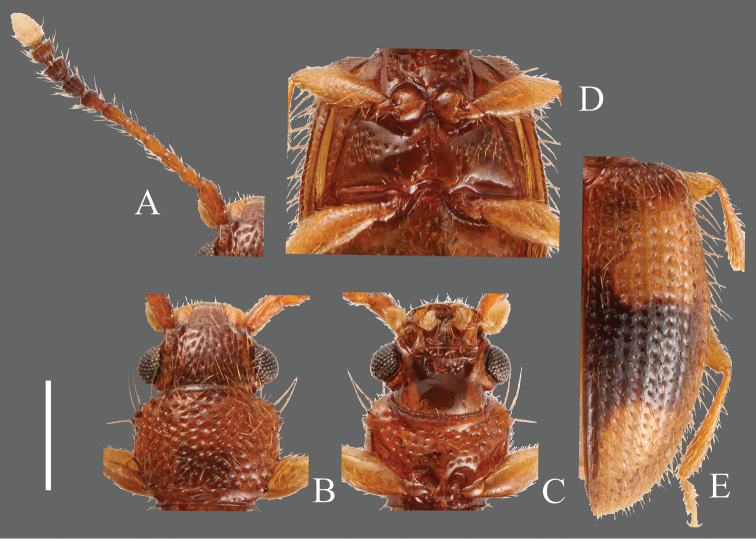
*Psammoecus scitus* sp. n., holotype, male. **A** Left antenna **B** head and pronotum of dorsal view **C** head and pronotum of ventral view **D** metaventrite **E** right elytron with rows of punctures and pubescence. Scale: 0.5 mm.

**Figures 4.**
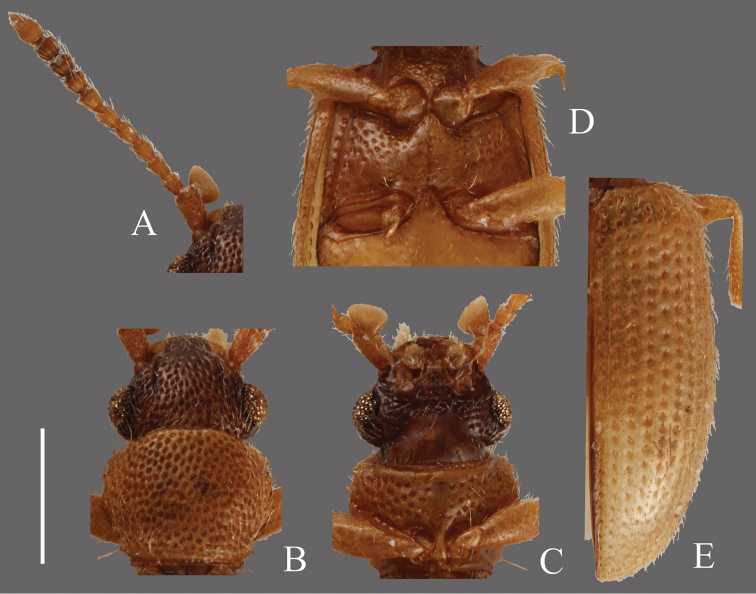
*Psammoecus bipunctatus* (Fabricius, 1792), male specimen which genital structures were illustrated in this paper. **A** Left antenna **B** head and pronotum of dorsal view **C** head and pronotum of ventral view **D** metaventrite **E** right elytron with rows of punctures and pubescence. Scale: 0.5 mm.

**Figures 5.**
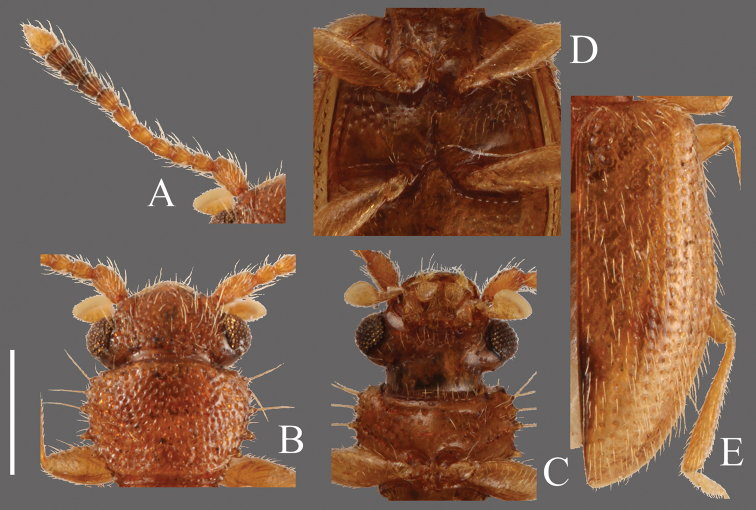
*Psammoecus trimaculatus* Motschulsky, 1858, male specimen which genital structures were illustrated in this paper. **A** Left antenna **B** head and pronotum of dorsal view **C** head and pronotum of ventral view **D** metaventrite **E** right elytron with rows of punctures and pubescence. Scale: 0.5 mm.

**Figures 6.**
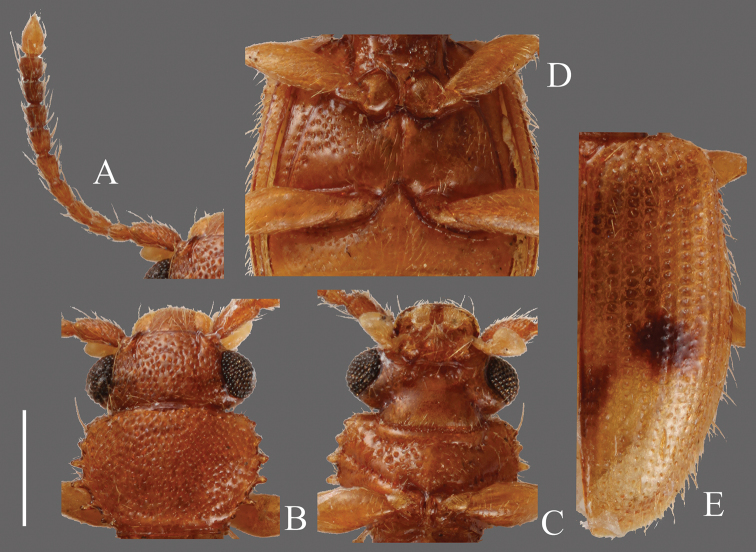
*Psammoecus triguttatus* Reitter, 1874, lectotype, male. **A** Left antenna **B** head and pronotum of dorsal view **C** head and pronotum of ventral view **D** metaventrite **E** right elytron with rows of punctures and pubescence. Scale: 0.5 mm.

**Figures 7.**
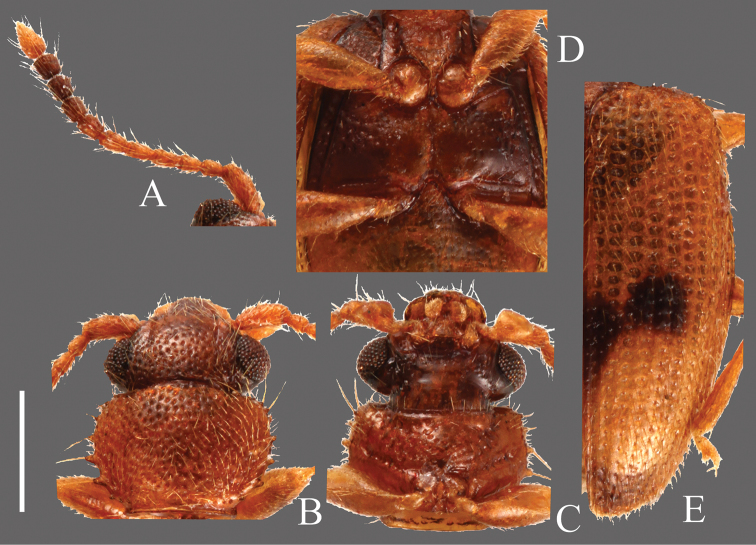
*Psammoecus labyrinthicus* sp. n., holotype, male. **A** Left antenna **B** head and pronotum of dorsal view **C** head and pronotum of ventral view **D** metaventrite **E** right elytron with rows of punctures and pubescence. Scale: 0.5 mm.

**Figures 8.**
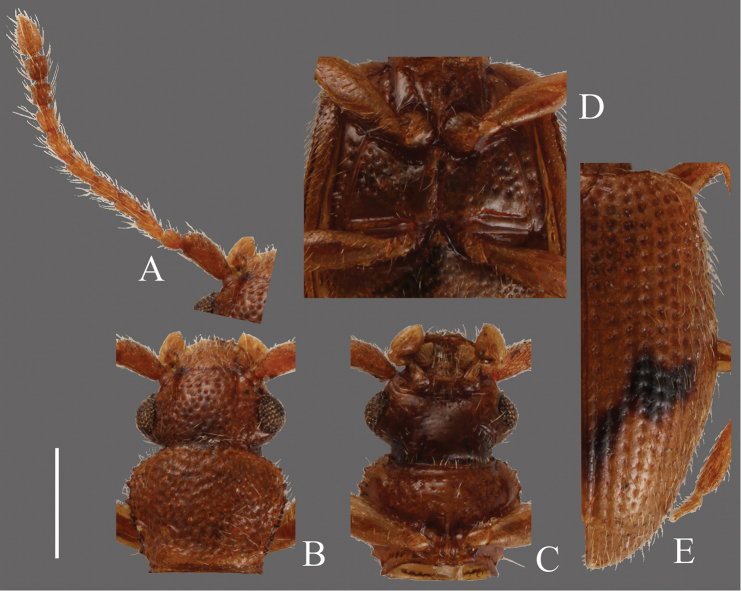
*Psammoecus boreas* sp. n., holotype, male. **A** Left antenna **B** head and pronotum of dorsal view **C** head and pronotum of ventral view **D** metaventrite **E** right elytron with rows of punctures and pubescence. Scale: 0.5 mm.

**Figures 9.**
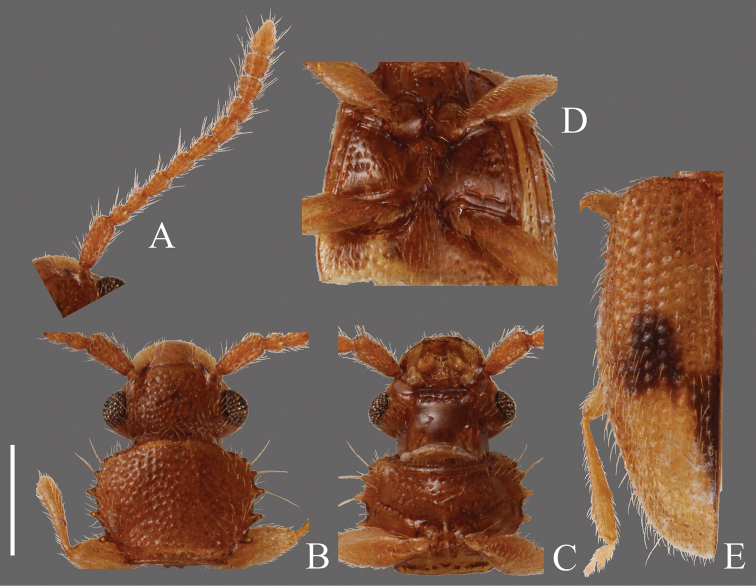
*Psammoecus omotoensis* sp. n., holotype, male. **A** Right antenna **B** head and pronotum of dorsal view **C** head and pronotum of ventral view **D** metaventrite **E** left elytron with rows of punctures and pubescence. Scale: 0.5 mm.

**Figures 10.**
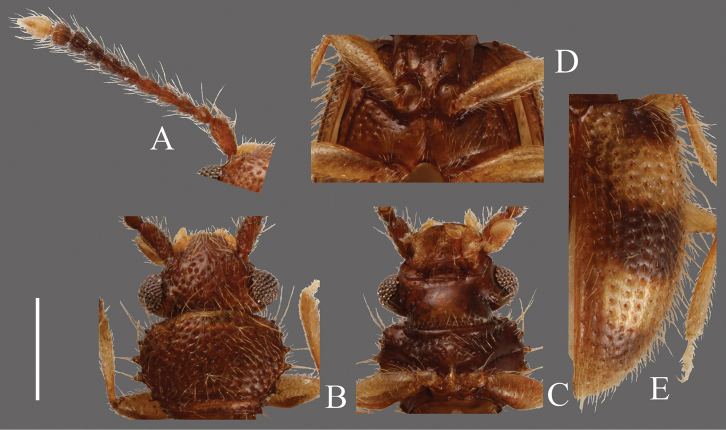
*Psammoecus simoni* Grouvelle, 1892, male specimen which genital structures were illustrated in this paper (**B–E**) and another male specimen (**A**). **A** Left antenna **B** head and pronotum of dorsal view **C** head and pronotum of ventral view **D** metaventrite **E** right elytron with rows of punctures and pubescence. Scale: 0.5 mm.

**Figures 11.**
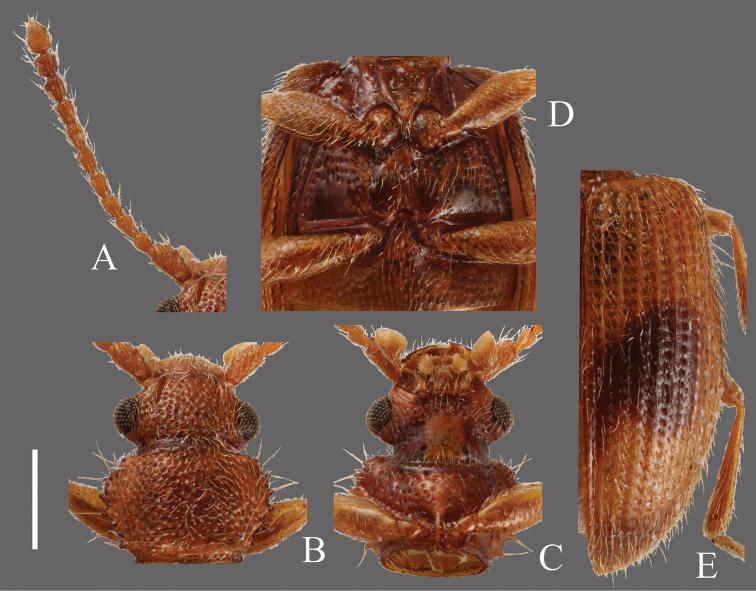
*Psammoecus fasciatus* Reitter, 1874, lectotype, male. **A** Left antenna **B** head and pronotum of dorsal view **C** head and pronotum of ventral view **D** metaventrite **E** right elytron with rows of punctures and pubescence. Scale: 0.5 mm.

**Figures 12.**
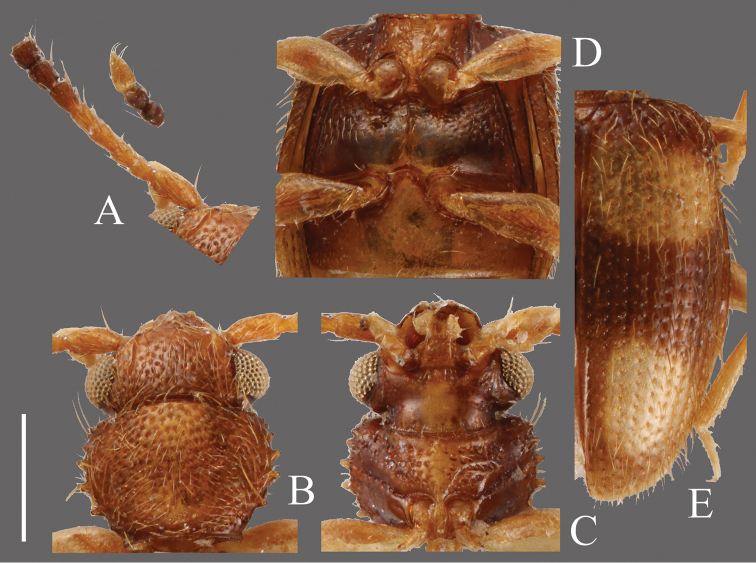
*Psammoecus quadrimaculatus* Reitter, 1874, lectotype, male. **A** Left antenna **B** head and pronotum of dorsal view **C** head and pronotum of ventral view **D** metaventrite **E** right elytron with rows of punctures and pubescence. Scale: 0.5 mm.

**Figures 13.**
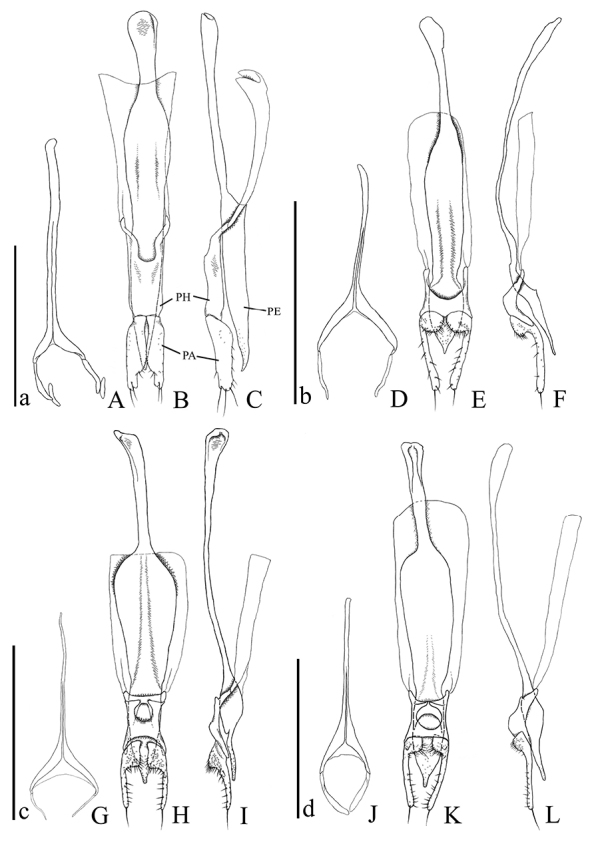
Male genital organs of *Psammoecus* spp. **A–C** *Psammoecus scitus* sp. n., holotype **D–F** *Psammoecus bipunctatus* (Fabricius, 1792) **G–I** *Psammoecus trimaculatus* Motschulsky, 1858 **J–L** *Psammoecus triguttatus* Reitter, 1874, lectotype. **A**, **D**, **G** and **J** 9th abdominal sternite **B**, **C**, **E**, **F**, **H**, **I**, **K** and **L** aedeagus in dorsal (**B**, **E**, **H** and **K**) and lateral (**C**, **F**, **I** and **L**). Abbreviations: PH—phallobase; PA—parameres; PE—penis. Scale: 0.5 mm; a for **A–C**; b for **D–F**; c for **G–I**; d for **J–L.**

**Figures 14.**
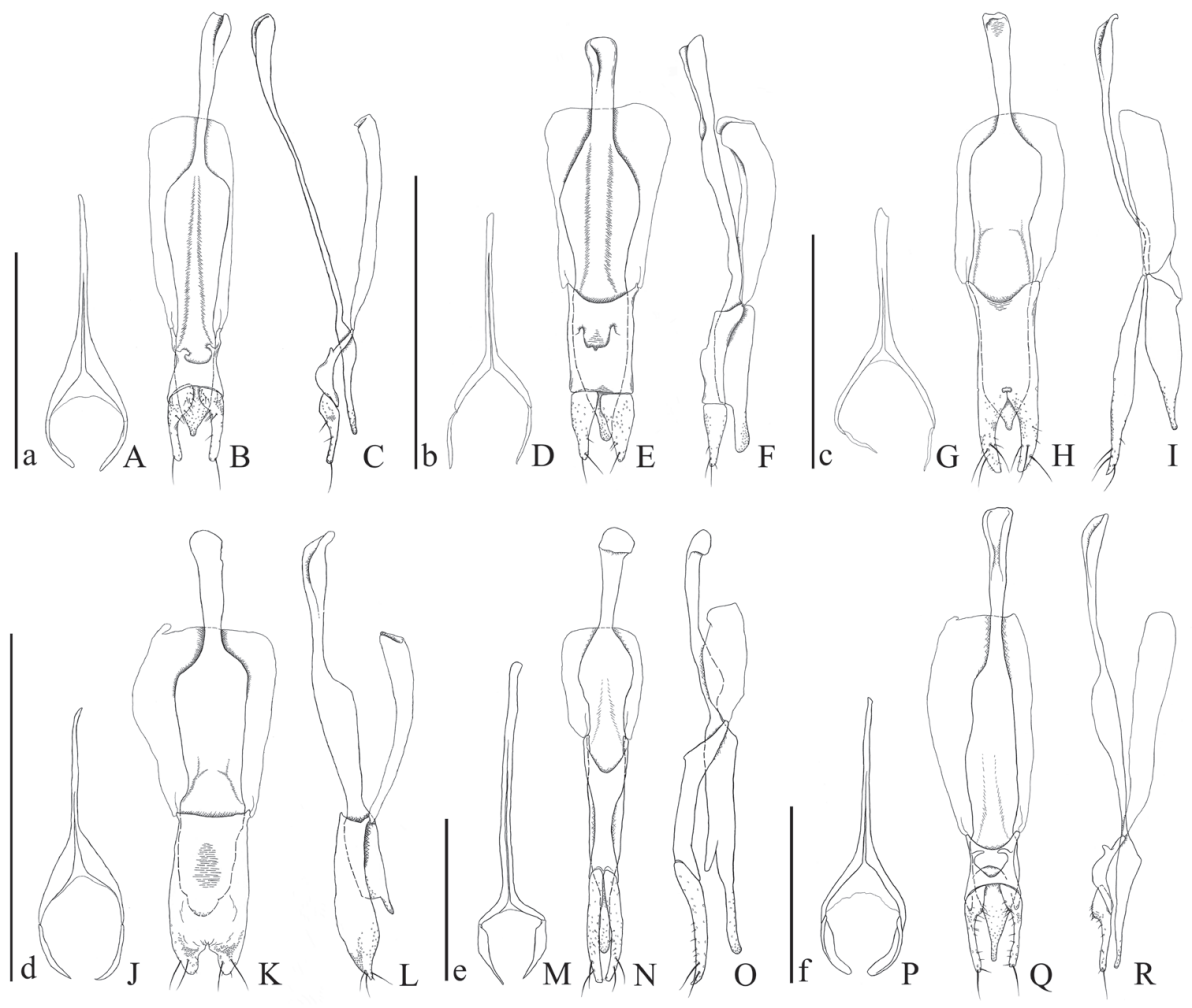
Male genital organs of *Psammoecus* spp. **A–C** *Psammoecus labyrinthicus* sp. n., holotype **D–F** *Psammoecus boreas* sp. n., holotype **G–I** *Psammoecus omotoensis* sp. n., holotype **J–L** *Psammoecus simoni* Grouvelle, 1892 **M–O** *Psammoecus fasciatus* Reitter, 1874, lectotype **P–R** *Psammoecus quadrimaculatus* Reitter, 1874, holotype. **A**, **D**, **G**, **J**, **M** and **P** 9th abdominal sternite; **B**, **C**, **E**, **F**, **H**, **I**, **K**, **L**, **N**, **O**, **Q** and **R** aedeagus in dorsal (**B**, **E**, **H**, **K**, **N** and **Q**) and lateral (**C**, **F**, **I**, **L**, **O** and **R**). Scale: 0.5 mm; a for **A–C**; b for **D–F**; c for **G–I**; d for **J–L**; e for **M–O**; f for **P–R.**

**Figures 15.**
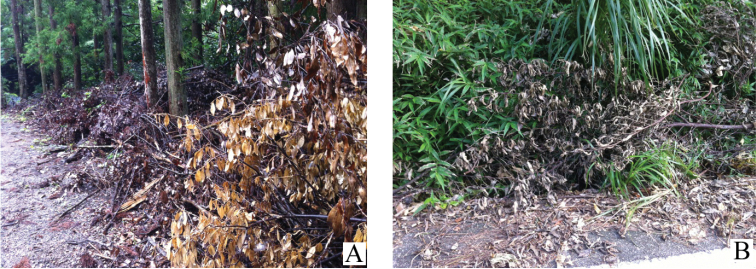
The habitat of *Psammoecus labyrinthicus* sp. n. (**A**) and *Psammoecus simoni* Grouvelle, 1892 (**B**) **A** Ta, Toyotama-chô, Tsushima Island, Nagasaki Prefecture, Japan **B** Inamine, Nago City, Okinawa Island, Okinawa Prefecture, Japan.

## Supplementary Material

XML Treatment for Psammoecus scitus

XML Treatment for Psammoecus bipunctatus

XML Treatment for Psammoecus trimaculatus

XML Treatment for Psammoecus triguttatus

XML Treatment for Psammoecus labyrinthicus

XML Treatment for Psammoecus boreas

XML Treatment for Psammoecus omotoensis

XML Treatment for Psammoecus simoni

XML Treatment for Psammoecus fasciatus

XML Treatment for Psammoecus hiranoi

XML Treatment for Psammoecus quadrimaculatus

## References

[B1] Blackburn T (1903) Further Notes on Australian Coleoptera, with Descriptions of New Genera and Species. Transactions and Proceedings and Report of the Royal Society of South Australia (incorporated) 27: 91-181

[B2] Fabricius JC (1792) Entomologia systematica emendata et aucta. Secundum classes, ordines, genera, species, adjectis synonimis, locis, observationibus, descriptionibus, Tom. 1. impresis Christ. Gottl. Proft, Hafniae, Copenhagen, 520 pp. doi: 10.5962/bhl.title.36532

[B3] Fabricius JC (1801) Systema elevtheratorvm secvndvm ordines, genera, species, adiectis synonimis, locis, observationibvs, descriptionibvs, Tomvs 1. impresis Bibliopolii Academici Novi, Kiliae, Germany, 506 pp.

[B4] Gerhardt J (1912) Neuheiten der schlesischen Koleopterenfauna aus dem Jahre 1911. Jahresheft des Vereins für schlesische Insektenkunde zu Breslau 5: 5-6

[B5] Grouvelle A (1892) Cucujides (in Voyage de M. E. Simon á l’îe de Luzon Philippines). Annales de la Société Entomologique de France 61: 285-288

[B6] Grouvelle A (1906) Contribution a l’étude des coléopteères de Madagascar Nitidulidae, Colydiidae, Cucujidae, Monotomidae, Cryptophagidae, Mycetophagidae, Dryopidae, Heteroceridae. Annales de la Société Entomologique de France 75: 67-144

[B7] Grouvelle A (1908) Coléoptères de la région Indienne. Rhysodidae, Trogositidae, Nitidulidae, Colydiidae, Cucujidae. Annales de la Société Entomologique de France 77: 315-495

[B8] Grouvelle A (1919) Descriptions d’espèces nouvelles du genre *Psammoecus* Latr. In: Mèmoires Entomologiques. Études sur les coléoptères. II. Société Entomologiques de France, Paris, 5–38

[B9] Grebennikov VV Leschen RAB (2010) External exoskeletal cavities in Coleoptera and their possible mycangial functions. Entomological Science 13: 81-98. doi: 10.1111/j.1479-8298.2009.00351.x

[B10] Halstead DGH (1986) Keys for identification of beetles associated with stored products. I-introduction and key to families. Journal of stored Products Research 22: 163-203. doi: 10.1016/0022-474X(86)90011-1

[B11] Halstead DGH Löbl I Jelínek J (2007) Silvanidae. In: Löbl I Smetana A (Eds) Catalogue of Palaearctic Coleoptera. Vol. 4. Elateroidea – Derodontoidea – Bostrichoidea – Lymexyloidea – Cleroidea – Cucujoidea Apollo Books, Stenstrup, 496-501

[B12] Hetschko A (1930) Fam. Cucujidae In: Coleopterorum Catalogus Pars 109. W. Junk, Berlin, 1–90

[B13] Hirano Y (2009) Notes on Japanese Silvanidae (Nihonsan hosohiratamushi-ka ni tsuite). Kanagawa-chûhô 168: 57-83 [In Japanese]

[B14] Hirano Y (2010) Cucujoidea of Japan Vol.2 Silvanidae, Byturidae, Biphyllidae. Roppon-Ashi Entomological Books, Tokyo, 61 pp. [In Japanese, with English title]

[B15] Hisamatsu S (1977) Some notes of clavicorn beetles from Hiroshima Prefecture (Hiroshima-ken san kyuukakugun kochu note). Hiroshima Mushi-no-kai Kaiho 16: 207-209 [In Japanese]

[B16] Hisamatsu S (1982) Some clavicorn beetles from Nepal (Coleoptera). Transaction of the Shikoku Entomological Society 16: 15-17

[B17] Kamiya H (1961) How to identify flat bark beetle (Nihonsan hiratamushi no miwake-kata). Tsukushi no Konchu 6: 15-18

[B18] Karner M (2012) A revision of African *Psammoecus* from the collection of the Musée royal de l’Afrique central. European Journal of Taxonomy 17: 1-31. doi: 10.5852/ejt.2012.17

[B19] Kreissl E (1976) Nachweise von Psammoecus bipunctatus (Fabr.) aus der Steiermark (Ins. Coleoptera, Cucujidae). Mitt. Abt. Zool. Landermus. Joanneum 5(1): 31-32

[B20] Latreille PA (1829) Suite et fin des insects In: Cuvier G (Ed) Le Regne Animal distribué d’après son Organization, pour server de base à l’histoire naturelle des animaux et d’introduction a l’anatomie comparée. Vol. 5 Déterville, Libraire, Paris, 556 pp.

[B21] Lawrence JF Beutel RG Leschen RAB Ślipiński A (2010) Glossary of morphological terms. In: Leschen RAB Beutel RG Lawrence JF (Eds) Handbook of Zoology, Coleoptera, Beetles, Vol. 2: Morphology and Systematics (Elateroidea, Bostrichiformia, Cucujiformia partim) Walter de Gruyter, Berlin New York, 9-20

[B22] Lawrence JF Ślipiński A Seago AE Thayer MK Newton AF Marvaldi AE (2011) Phylogeny of the Coleoptera based on morphological characters of adults and larvae. Annales Zoologici (Warszawa) 61: 1-217. doi: 10.3161/000345411X576725

[B23] Lu Y Han Z (2006) Five narrowly distributed species of Silvanidae from Yangzhou captured in wet blue leather and packages. Chinese Bulletin of Entomology 43: 398-400 [In Chinese, with English title]

[B24] Lucas PH (1843) Note sur quelques nouvelles espèces d’insectes de la famille des Trachélides qui habitent les possessions françaises du nord de l’Afrique. Revue Zoologique, par la Société Cuvierienne; Association Universelle pour l’Avancement de la Zoologie, de l’Anatomie Comparée et de la Palaeontologie; Journal Mensuel. Publié sous la Direction de M.F.-E. Guérin-Méneville 7: 145-147

[B25] Nakane T (1963) Silvanidae In: Nakane T Ohbayashi K Nomura S Kurosawa Y (Eds) Iconographia Insectorum Japonicorum Colore naturali edita. Vol. 2 (Coleoptera), Hokuryukan, Tokyo, 195–196 (incl. pl. 98) [In Japanese]

[B26] Pal TK (1985) A revision of Indian *Psammoecus* Latreille (Coleoptera: Silvanidae). Records of the Zoological Survey of India 71: 1-54

[B27] Reitter E (1874) Beschreibungen neuer Käfer-Arten nebst synonymischen Notizen. Verhandlungen der Kaiserlich-Königlichen Zoologisch-Botanischen Gesellschaft in Wien 24: 509-528

[B28] Reitter E (1879) Beitrag zur Synonymie der Coleopteren. Verhandlungen der Kaiserlich-Königlichen Zoologisch-Botanischen Gesellschaft in Wien, 29, 507–512

[B29] Sasaji H (1985) Silvanidae In: Kurosawa Y Hisamatsu S Sasaji H (Eds) The Coleoptera of Japan in Color Vol. III Hoikusha Publishing, Osaka, 202–205 (incl. pl. 32) [In Japanese, with English title]

[B30] Sasaki T Kimura M Kawamura F (2002) Coleoptera. In: Azuma S (Ed) Check List of the Insect of the Ryukyu Islands (Second Edition). The Biological Society of Okinawa, Nishihara, 157-284 [In Japanese, with English title]

[B31] Sato M (1989) Coleoptera. In: Hirashima Y (Ed) A Check List of Japanese Insects I Entomological Laboratory, Faculty of Agriculture, Kyushu University, Fukuoka, 197-538 [In Japanese]

[B32] Schaufuss C (1916) Calwer’s Käferbuch Einführung in die Kenntnis der Käfer Europas. Band I. E. Schweizerbart’sche Verlagsbuchhandlung, Stuttgart, 709 pp.

[B33] Smith F (1851) Catalogue of the Cucujidae, &c. List of the Coleopterous Insects in the Collection of the British Museum 1: 1-25

[B34] Thomas MC (1993) The flat bark beetles of Florida (Coleoptera: Silvanidae, Passandridae, Laemophloeidae). Arthropods of Florida and Neighboring Land Areas 15: 1–93 http://ufdc.ufl.edu/UF00000095/00001

[B35] Thomas MC (2002) Silvanidae Kirby 1837. In: Arnett RH Thomas MC Skelley PE Frank JH (Ed) American beetles. Vol. 2. Polyphaga: Scarabaeoidea through Curculionoidea CRC Press, Boca Raton, 322-326

[B36] Thomas MC Nearns EH (2008) A new genus of telephanine Silvanidae (Coleoptera: Cucujoidea), with a diagnosis of the tribe and key to genera. Insecta Mundi 0048: 1–14 http://digitalcommons.unl.edu/insectamundi/576

[B37] Thomas MC Leschen RAB (2010) Silvanidae. In: Leschen RAB Beutel RG Lawrence JF (Eds) Handbook of Zoology, Coleoptera, Beetles, Vol. 2: Morphology and Systematics (Elateroidea, Bostrichiformia, Cucujiformia partim) Walter de Gruyter, Berlin New York, 346-350

[B38] Thomas MC Yamamoto PT (2007) New records of Old World Silvanidae in the New World (Coleoptera: Cucujoidea). Coleopterists Bulletin 61: 612-613. doi: 10.1649/0010-065X(2007)61[612:NROOWS]2.0.CO;2

[B39] Walker F (1859) Characters of some apparently undescribed Ceylon Insects. The Annals and Magazine of Natural History, including Zoology, Botany, and Geology 3(3): 50-56

[B40] Warne AC (1963) The insects of Thriplow meadows. Nature in Cambridgeshire 6: 24–26 http://www.natureincambridgeshire.org.uk/volumes/nature-in-cambs-vol-06-1963.pdf

[B41] Waterhouse CO (1876) Descriptions of new Cucujidae and Cleridae. The Entomologist’s Monthly Magazine 13: 118-126

[B42] Yoshida T Hirowatari T (2013) A New Species of the Genus *Psammoecus* (Coleoptera, Silvanidae) from the Nansei Islands, Japan. Japanese Journal of Systematic Entomology 19: 85-90

